# Single Molecule Electronics and Devices

**DOI:** 10.3390/s120607259

**Published:** 2012-05-30

**Authors:** Makusu Tsutsui, Masateru Taniguchi

**Affiliations:** The Institute of Scientific and Industrial Research, Osaka University, 8-1 Mihogaoka, Ibaraki, Osaka 567-0047, Japan; E-Mail: makusu32@sanken.osaka-u.ac.jp

**Keywords:** single-molecule electronics, electron-phonon interaction

## Abstract

The manufacture of integrated circuits with single-molecule building blocks is a goal of molecular electronics. While research in the past has been limited to bulk experiments on self-assembled monolayers, advances in technology have now enabled us to fabricate single-molecule junctions. This has led to significant progress in understanding electron transport in molecular systems at the single-molecule level and the concomitant emergence of new device concepts. Here, we review recent developments in this field. We summarize the methods currently used to form metal-molecule-metal structures and some single-molecule techniques essential for characterizing molecular junctions such as inelastic electron tunnelling spectroscopy. We then highlight several important achievements, including demonstration of single-molecule diodes, transistors, and switches that make use of electrical, photo, and mechanical stimulation to control the electron transport. We also discuss intriguing issues to be addressed further in the future such as heat and thermoelectric transport in an individual molecule.

## 1. Introduction

**Figure 1.**
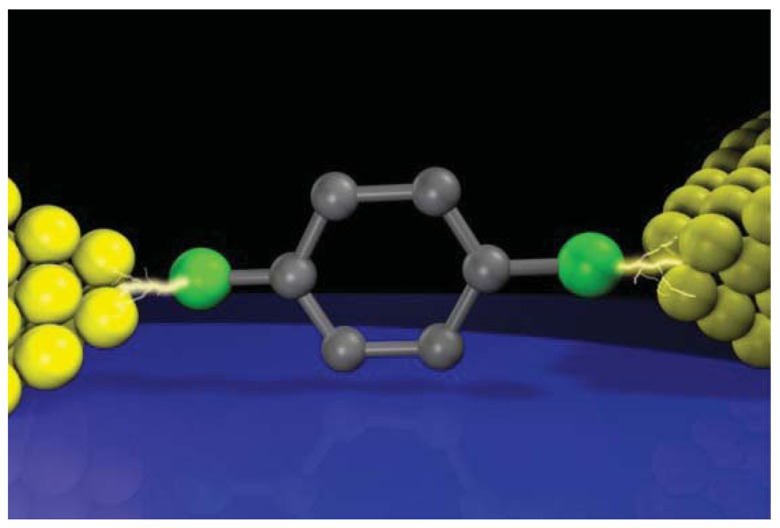
Schematic model of a single-molecule junction.

Miniaturization of electronics devices has been progressed rapidly owing to the advanced silicon technology for integrating billions of silicon-based building blocks in a millimeter-scale chip. Ultimate downscaling of active electronic components such as diodes and transistors can be achieved by making use of electron and hole transport in single organic molecules connected to two electrodes (Figure 1).

This attractive idea is called molecular electronics (or single molecule electronics) and has been actively pursued since Aviram and Ratner proposed the first basic design of molecular rectifier in 1974 [1]. Unlike the energy-band dispersion relation of bulk materials, the energy levels are quantized in an individual molecule anchored to electrodes. Electron transport in this single-molecule junction is characterized by charge injection barriers at the electrode-molecule interfaces, which is determined by energy alignment between the electrode Fermi level and a single discrete energy level of the molecule; either the highest occupied molecular orbital (HOMO) or the lowest unoccupied molecular orbital (LUMO) levels. Electronic coupling of the current-carrying individual molecules with the macroscopic electrodes via the overlaps of molecular wavefunction and that of the electrodes also affects the charge transmissivity by broadening the frontier orbital levels [2]. Significant efforts have been devoted to achieve modulation of the Fermi alignment to the HOMO (LUMO) and/or the molecule-electrode coupling with external stimulus such as electric field, light, and mechanical forces, for understanding and controlling the unique electrical characteristics of single-molecule junctions.

While physical properties of organic thin films have already been well-studied and proven to be useful in industries as flexible electroluminescent devices for multicolor display applications, however, single-molecule electronics is an emerging field because only recently has it become possible to form stable metal/single-molecule/metal systems and identify the molecular signatures to ensure their existence in the electrode gap. In this review, we summarize the technical approaches to form single-molecule junctions with an emphasis on their advantages for fabricating stable molecular bridges. It also covers essential spectroscopic techniques now incorporated in many single-molecule electron transport measurements that exploit inelastic electron-phonon interactions for revealing the presence of a target molecule in the electrode gap. We then introduce recent remarkable experimental progress in demonstrating single-molecule device functionalities including diodes, field-effect transistors, and mechanical-switches. In the final part of this paper, we discuss the potential use of single-molecule thermoelectric devices.

## 2. Experimental Techniques for Forming Single-Molecule Junctions

Investigation of single-molecule electron transport calls for a reliable and efficient method for constructing metal/single-molecule/metal system. Early measurements on molecular junctions have discovered many intriguing phenomena such as the Kondo effect, negative differential resistance, and Coulomb blockade. In these works, various apparatus have been utilized to form meta/molecule/metal structures such as electromigrated nanogaps [3–5], scanning probe microscopy (SPM) [6,7], nanopore devices [8], and mechanically-controllable break junction [9]. However, interconnecting just one molecule to two electrodes has been formidable task due to the lack of information available in the experiments to estimate the number of current-carrying molecules in the gap.

**Figure 2.**
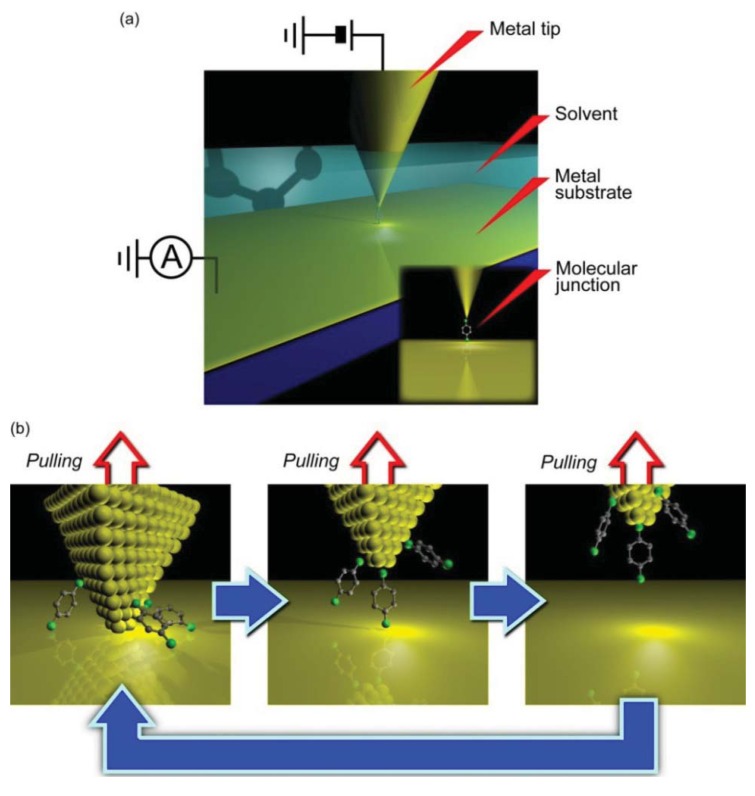
SPM break junction method. (**a**) The system consists of a metal substrate covered with self-assembled monolayer of a target molecule and a metal tip; (**b**) A tip is indented into a substrate in a solvent. Subsequently, the fused contact is stretched by pulling out the tip and the conductance trace is measured (left). In case of Au junctions, the conductance drops in a stepwise fashion and show a long plateau at 1 *G*_0_ signifying formation of Au single atom chains. After breaking the Au contacts, current flows through several molecules bridging the tip and the substrate. Further retracting the tip, the metal-molecule bonds rupture and the number of the current-carrying molecules decreases one by one showing conductance staircases (middle) and finally to zero (right). Thousands of single-molecule junctions can be formed within a relatively short time by repeating the series of processes, which allows deduction of junction-to-junction variations of the single-molecule conductance.

Discussion about single-molecule conductance has become possible by applying a repeated break junction approach (Figure 2), which was widely utilized for studying electrical properties of atomic-sized contacts [10,11]. In this method, two free-standing metal electrodes are mechanically moved into and out of contact to each other repeatedly. The junctions undergo necking deformation and thinned gradually when pulled apart. Eventually the two electrodes become hanged with only several atoms. Further stretching of the atomic-sized constriction leads to a decrease in the number of the contact atoms. As a result, a single-atom wire is formed right before the junction breakdown [10–13]. These processes can be observed as a stepwise decrease of the conductance to zero in response to the mechanical stretching and concomitant atom rearrangements in the contact. Because the size of metallic single-atom contacts is comparable to the Fermi wavelength, their conductance is described by the Landauer expression *G* = 2*e*^2^/*h*Σ*T_i_*, where *T*_i_ is the transmission probabilities in the conductance channels. The number of channels is determined by the number of orbitals. It is known that monovalent metal single-atom contacts (such as Au, Cu, and Ag) possess a fully opened single channel for electron transmission that gives contact conductance of 1 *G*_0_, where *G*_0_ = 2*e*^2^/*h* is the conductance quantum [10,11]. This allows rough estimation of the contact size at the single atom level; a contact with *G* = *nG*_0_ is comprised of *n* atoms. By analogy to this method, single molecule counting was examined by Tao *et al.* performing the break junction measurements in a solution containing organic molecules using a SPM set up [14]. In this case, the molecules were allowed to bridge between the Au electrodes after junction breakdown. As a result, conductance staircase was observed at *G* < 1 *G*_0_ suggesting successive disconnection of the current-carrying molecules as the electrode gap distance was increased. Corresponding conductance histograms constructed with hundreds or sometimes thousands of conductance traces revealed several peaks at *G* = *nG*_SMC_ (n = 1, 2, 3,…), which could be naturally assigned to the conductance states of metal/molecule/metal system consisting of *n* molecules connected in parallel to the electrodes with the single-molecule conductance of *G*_SMC_ [14]. This simple yet powerful approach for statistical evaluation of electrical conductance of single-molecule junctions has spurred a large number of studies, both theoretical and experimental, on single-molecule electron transport in various kinds of organic molecules and electrode metals [15–20]. In this section, we review three types of break junction techniques used for fabricating single-molecule junctions: SPM break junction, micro-fabricated mechanically-controllable break junction (MCBJ), and electromigration break junction (EBJ).

### 2.1. SPM Break Junction

SPM break junction is probably the most widely used method for studying electron transport in single-molecule junctions. It usually utilizes scanning tunneling microscope or atomic force microscope facilities (Figure 2(a)). In the experiments, a macroscopic hydrogen-flame-annealed metal tip is indented into a metal-coated substrate electrode onto which a self-assembled monolayer is prepared and subsequently withdrawn (Figure 2(b)) [14,21]. The tip motion is often feedback controlled via voltage applied to the piezo-actuator for better reproducibility of the data [22]. The junction stretching speed during conductance trace measurements is defined by the sweep rate of the piezo-voltage, and is typically several tens of nanometer per second. The conductance trace measurements in most of the SPM break junction experiments were implemented in a liquid environment by placing the tip and the substrate in a Teflon cell filled with solvent.

The SPM approach has contributed considerably to advancing our understanding of electron transport in single molecules. Measurements on alkanedithiols have revealed an exponential decrease in the single-molecule conductance with the number of methylenes in the backbone that manifested tunneling electron transport through the saturated molecules [14,23–27]. Exponential decay of the conductance with the molecular length has also been reported for various π-conjugated molecules, e.g., oligophenylenes [28], oligopeptides [29], caltenoid polyenes [30], oligothiophenes [31], and oligo(phenylene ethinylene) [32], and these molecules showed smaller tunneling decay constant owing to the role of delocalized HOMO and LUMO. A tunneling to hopping transition of charge conduction through a molecular wire was also confirmed by extending the measurements to long length molecules [32–34]. While these results reported smaller conductance for longer molecules, peculiar length dependence has been observed for short oligothiophenes, where terthiophenes exhibited lower single-molecule conductance than tetrathiophenes [35]. This was attributed to a smaller charge injection barrier for tetrathiophenes having HOMO levels closer to the Au electrode Fermi level. The role of molecular orbital levels on single-molecule tunneling conductance has been demonstrated by beautiful experiments by Venkataraman *et al.* [36]. They used many types of substituent molecules that donate or withdraw electrons to raise (lower) or lower (raise) the HOMO (LUMO) of benzenediamines, and find that the single molecule conductance increases with donor-like substituents, thereby suggested dominant role of HOMO on the conductance [36].

Besides the Fermi alignment issue, a suitable anchoring group for developing highly conductive and stable molecular junctions has been pursued, which is important from viewpoint of electronics device applications. Thiol (SH) has been the most frequently employed anchor group because one can exploit the strong Au-S bonds to form a stable metal-molecule-metal structure when using Au electrodes. However, the wide variation in the Au-S contact motif, whether it binds to top, bridge, or hollow sites of Au surface, gives rise to a broad distribution of the single-molecule conductance not suitable for device applications [37–39]. Furthermore, thiol was theoretically predicted to not be an optimal choice from the viewpoint of contact transparency [37], which was later corroborated by a photoelectron spectroscopy study on a monolayer [40–42]. Thus, to find metal-molecule links with superior stability and electronic coupling than Au-S linkages, many kinds of anchoring groups have been tested including pyridine [14]], amine [26,43,44], carboxylic acid [23], isocyanide [45,46], phosphine [24], selenol [47,48], and even C_60_ [49] using archetypical molecule wires such as alkanes and phenylenes. Amine (NH_2_) is found to provide more well-defined metal-molecule contact geometries because it can only be bonded to atop sites via its lone pair [26,43,44]. This idea was also applied to thiols; the single-molecule conductance distribution became narrowed by replacing thiols with methyl thiols [24], which was ascribed to the selective metal-molecule contact configurations for bonding between the sulfur lone pair of methyl thiols and the undercoordinated atop Au atoms on the electrode surface. Although incorporation of methyls to saturate the anchor units is proven to be effective approach for restricting the contact configuration variations, it also resulted in weaker metal-molecule bonds, which should be avoided for the sake of building robust single-molecule devices [50–52]. Recently, amazing results were reported by Cheng *et al.* [53]. They used Au break junctions and measured single-molecule conductance of alkanes with both ends terminated with trimethyl tin groups. It was found that the trimethyltin units tend to dissociate on the Au surface and at the same time the alkane chain creates covalent Au-C sigma bonds at both the ends. This new type of metal-molecule bonding design offered single alkanes with 100 times higher conductance compared to the other conventional anchors together with a narrow conductance distribution and excellent bond strength of 3.0 eV [53].

The careful and systematic SPM break junction experiments have led to the advance in understanding and control of electron transport in single molecules and provided a useful guideline for constructing robust and highly conductive molecular junctions. Beyond this, there is growing interest in using SPM to construct and realize single-molecule device concepts such as single molecule diodes and transistors. However, thermal drift poses a critical difficulty in holding molecular bridges for more than 10 s at room temperatures, and such research is often possible only under cryogenic vacuum conditions. In this sense, EBJ and MCBJ techniques have advantages in that they possess superior mechanical stability as described below.

### 2.2. Micro-Fabricated MCBJ

**Figure 3.**
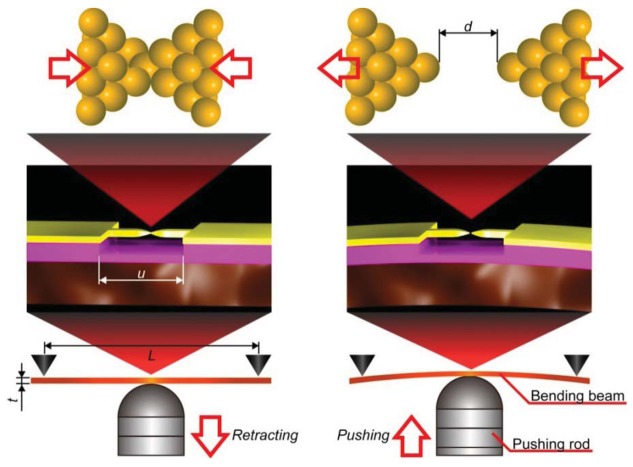
Principle of operation of micro-fabricated MCBJs. The MCBJ set up (bottom) is comprised of a free-standing metal junction formed on a polyimide-coated phosphor bronze substrate using electron beam lithography and metal deposition processes (middle). The narrowest constriction at the center is usually sub-micrometer size. The phosphor bronze substrate is deflected by the pushing rod in a three-point bending configuration, which induces the tensile force on the free-standing junction to break it. After breaking, an electrode gap of size *d* is formed (top). Thereafter, the electrode gap distance can be finely tuned by the vertical displacement of the pushing rod *D* by *d* = *rD*, where *r* is the attenuation factor roughly determined by the device configuration as *r* = 3*ut*/*L*. Usually, *r* is estimated by measuring the exponential decay of the current flowing across the electrode gap with increasing *D* in a vacuum.

MCBJ consists of a free-standing metal wire fixed on a flexible beam (Figure 3) [11]. Usually, phosphor bronze plate coated with thick polyimide layer is used for the substrate. As the name implies, the principle of MCBJ is essentially the same as that of SPM break junction approach; mechanical manipulation of distance between the two facing electrodes to form a metal/molecule/metal system. The difference lies in the way we move the electrodes; whereas SPM directly moves the tip with piezo-actuator displacements, MCBJ makes use of substrate deflection caused by piezo-driven pushing rod motion to indirectly displace the positions of two free-standing electrodes on the substrate (Figure 3) [11]. The ingenious structure of MCBJ enables fine tuning of the inter-electrode distance. The pushing rod displacement is translated into the electrode displacement with the attenuation factor *r* [11,54]. Micro-fabricating the metal nanobridge, *r* can be made smaller than 10^−4^, which allows control of the electrode displacement at sub-picometer resolution by nanometer-level manipulation of the pushing rod displacements with a piezo-element [11,55,56]. At the same time, mechanical loop of micro-fabricated MCBJs, defined by the length of the nanobridge, is reduced to several micrometers. This amazingly short mechanical loop gives outstanding mechanical stability necessary for forming and holding single-molecule junctions for prolong time at room temperatures [11].

In addition to their applications for studying single-molecule electron transport, the unique capability of micro-fabricated MCBJs of adjusting the electrode gap distance at sub-picometer precision has been utilized for studying the stability and current carrying capacity of individual organic molecular wire at room temperatures [57,58]. In these works, single-molecule junctions were repeatedly formed and broken by the break junction method with a slow junction elongation speed down to 0.6 pm/s.

Special care was taken to avoid possible effects of Au nanocontact thinning processes on the molecular junction stability and meanwhile to optimize the production efficiency [59,60]. For this purpose, the Au junction stretching speed was multistep feedback controlled in the pre-breakdown stage [59,60]. This automated metal/molecule/metal structure fabrication method was recently exploited in an interesting break junction experiments using a STM system customarily designed to suppress mechanical drift [61]. Because the molecular junction breakdown is a thermally activated stochastic event, the holding duration, or the lifetime, of single-molecule bridges is described in Arrhenius form as:

(1) $$\tau =\left(1/f\right)\exp\left[\left(E_{\text{B}}-\alpha F-\beta V_{\text{b}}\right)/k_{\text{B}}T_{\text{eff}}\right]$$

where *f, E*_B_, *αF, βV*_b_, *k*_B_, and *T*_eff_are the attempt frequency, the barrier energy for junction breakdown, the mechanical tensile forces [62,63], the current-induced forces [64–66], Boltzmann factor, and the contact temperature including the local electrical heating effects [67–70]. In the experiments, *τ* could be deduced from the duration of sustaining the single-molecule conductance state during junction breaking [57]. The breakdown mechanism was found to be classified into three different regimes with respect to the displacement speed [59]. At high strain rates, the tensile force exerted on both sides of the contact during elastic deformation dominates, and it tends to dissociate under a certain amount of force, as has also been observed in SPM break junction measurements [68,71]. When the elongation speed is lowered, effects of thermal fluctuations become non-negligible, and the force necessary to break the junction decreases logarithmically to zero with decreasing stretching rates [59]. The natural lifetime can be assessed either indirectly by extrapolating the stretching rate dependence of the junction lifetime to zero breakdown force or directly under a slow-enough elongation rates whereas contribution of thermal fluctuations becomes predominant and junction breaks spontaneously [58]. For Au single-atom contacts, the breakdown force approaches zero at the stretching rate below 6 pm/s. The room temperature average contact lifetime at this low strain rate regime took a constant value of about 10 s, suggesting self-breaking of a Au-Au link with a bonding energy of 0.8 eV under negligible influence of the mechanical forces [59]. Benzenedithiol single-molecule junctions were found to be much more stable, as expected from the strong Au-S binding energy of 1.6 eV, with the natural lifetime of 50,000 s [58]. However, this junction lifetime was also too short for Au-S bonds to dissociate thermally at room temperatures, suggesting contact breakdown at the Au-Au bonds in the bank. In contrast, benzenediamines can be hold for 0.2 s on average, shorter lived than Au single atom contacts [51]. This clearly indicates that the diamine molecules rupture at the NH_2_-Au links with bond energy of 0.7 eV. These findings not only shed light on the formation and breakdown mechanisms of molecular junctions, but also prove the potential of micro-fabricated MCBJs for quantitatively evaluating the single-molecule device stability [51].

The self-breaking method to rupture molecular bridges without external mechanical forces also offers a platform useful for studying heat dissipation [67–69,72]. Under the high-field, electron-phonon interactions induce considerable local heating in molecular conductors [68,69,72]. Understanding and controlling the electrical heating mechanism is an important issue as it strictly limits the current carrying capacity of single-molecule devices. The rise in the effective temperature *T*_eff_ of single-molecule junctions can be estimated by examining the lifetime decay with the applied voltage using Equation (1). In SPM break junctions, substantial influence of external forces on the junction lifetime is inevitable as thermal drift becomes problematic under slow stretching rate conditions [73]. This complicates the *T*_eff_ deduction from the analyses of the junction holding time. In contrast, the zero-force conditions attained in the self-breaking approach simplifies the description of the molecular bridge lifetime as *τ* = (1/*f*)*exp*[*E*_B_/*k*_B_*T*_eff_]. The voltage dependence of *T*_eff_ deduced in this way for Au/single-benzenedithiol/Au junctions [58] revealed effective temperature increases of up to 170 K from the ambient temperature at *V*_b_ = 1 V with *T*_eff_ ∼ (*V*_b_)^1/2^ as derived theoretically [68]. On the other hand, the local temperature of Au single atom contacts measured under the same conditions showed a negligible increase in *T*_eff_ demonstrating marginal influence of local heating, which was attributed to the effective lattice cooling by heat conduction to the bulk [66,74]. This manifested a large thermal resistance at the Au-benzenedithiol contacts due to the phonon mismatch between the high energy optical vibration modes of benzenedithiols and the low energy acoustic phonons of the Au electrodes that impedes heat dissipation from the junction to the electrodes [68]. As it is difficult to dissipate the local heat generated in the junctions vertically through the substrate, which is the strategy applicable for graphene [75] and carbon nanotube devices [76], development of phonon-transparent electrode-molecule contacts is perhaps essential for achieving high current carrying capacity of single-molecule bridges.

### 2.3. Electromigration Break Junction

**Figure 4.**
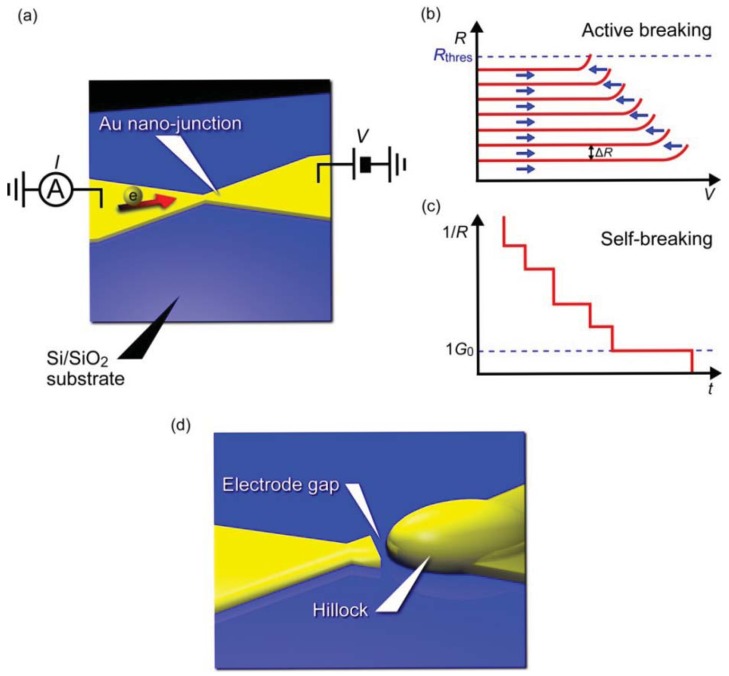
Electromigration break junction method. (**a,b**) During active breaking stage, the bias voltage applied on a Au nano-junction formed on a SiO_2_/Si wafer is feedback controlled to prevent overcurrent melting that results in formation of a wide electrode gap. Specifically, *V* is increased linearly until the contact resistance decreases by Δ*R* via electromigration thinning and Joule heating, and thereafter the bias sweep is restarted from a low voltage; (**c**) When the junction is thinned to atomic size, it is let to break spontaneously under a constant low bias voltage. In this self-breaking regime, the conductance decreases in a stepwise manner. A plateau often appears at near 1 *G*_0_ right before the formation of an electrode gap, which suggests formation of a Au single atom contact; (**d**) These two-step electrical breaking processes allow formation of a nano-meter electrode separation without involving nucleation of Au nanoparticles. A hillock is formed at the current downstream by electromigration of contact atoms.

Pioneering studies on three-terminal single-molecule devices utilized electromigration to form two electrodes separated by a nanometer gap for constructing molecular bridges [3–5,77]. This fascinating idea can be imagined as an electrical version of a mechanical break junction approach replacing the mechanical forces with electron wind forces (Figure 4). When a voltage ramp is applied to a lithographically defined metal nanowire, it ruptures at the nano-constriction under the huge current density that induces Joule-heat-activated electromigration of the contact atoms [78,79]. Usually, a self-assembled monolayer is prepared on the metal wire. The molecules are thermally diffused on the surface during the electrical breaking and a few of them become trapped in the electrode gap by chance, whereby a metal/molecule/metal structure is formed. There are several technical advantages in the electromigration break junction approach. Most importantly, the planar device architecture is compatible with silicon technology and amenable for integrating additional functionality such as electronic gating using a three-terminal device configuration. The electrode gap defined by the solid-state nanoelectrodes on the substrate is also highly mechanically stable. On the contrary, the gap distance is not controllable in the post-breaking stage unlike the case for mechanical break junctions that allow adjustment of the electrode positions to form a gap of arbitrary size. Therefore, the fabrication yield of molecular junctions is often very low. Moreover, metal nano-clusters are nucleated during electromigration processes, which are then trapped in the electrode gap and behave like molecules by creating parasitic electron conduction pathways and showing Coulomb blockade and Kondo effects that mimic electrical characteristics of single-molecule junctions [80–82].

Recently, improvements have been introduced to the original electromigrated gap formation method for achieving better reproducibility and yield [83–85]. Instead of using a single ramp to rapture a metal nano-junction, the bias voltage is feedback controlled by decrease in the conductance associated with the electromigration thinning (Figure 4(a,b)) [83]. This enables regulation of the self-accelerating nature of the electromigration failure processes and prevents the junction from undergoing overcurrent breakdown leading to considerably large electrode gaps. The active electrical breaking proceeds until the nano-constriction evolves into atomic size wire, after then the junction is left to break spontaneously by thermal fluctuations under a low voltage (Figure 4(c)). Electrode gaps fabricated in this way can be made as small as 1 to 2 nm (Figure 4(d)). Perhaps, the most important benefit of employing this self-breaking technique is the fact that the somewhat gentle way of contact breaking does not involve formation of metal nanoparticles [84,85].

It is important to understand the underlying physics of the electromigration junction breakdown. Electrical breaking of metallic nanocontacts occurs via the current-induced forces and the local heating. Which factor becomes critical depends on material. It is predicted theoretically that contact tends to break by local heating when the heat cannot be removedefficiently from the contact to the bulk leads by thermal conduction [86]. On the other hand, when the lattice cooling is effective, the contact breaks under high current density by the current-induced forces [58,74,86]. Systematic studies should be carried out to shed light on this intriguing subject. Although the electromigration-induced wire breaking approach lacks the controllability of the electrode gap distance, the planar solid-state electrode architecture is more attractive than the mechanical break junction methods from view point of large-scale integration of arrays of single-molecule devices, and may find future applications not only in molecular electronics but also as a biosensing platform in the emerging field of nanopore sequencing [87,88].

In fact, three-terminal single-molecule device concepts have long been explored using the electromigration break junction method. In these experiments, identification of the molecule species in the electrode gap is crucial for dealing with the nanoparticle nucleation issue. In this context, increasing effort has been devoted to incorporate spectroscopic techniques such as Raman spectroscopy and inelastic electron tunneling spectroscopy (IETS) in break junction experiments, which serve to ensure the presence of target molecules between the electrodes thereby improving the measurement data reliability as summarized below.

### 2.4. Diagnostic Tools for Identifying Molecular Species in Electrode Gap

Mechanical break junction methods have proven to be a useful tool for determining the electrical conductance of molecular tunneling bridges at the single-molecule level by the fact that theoretical calculations of the electron transport in molecular junctions in the elastic tunneling regime can now quantitatively explain the break junction measurements [17]. Beyond the fundamental studies on single-molecule electron transport, active control of the electronic properties is pursued for molecular electronics devices [16,19]. Owing to the various techniques developed for fabricating metal/molecule/metal systems (e.g., mechanical and electromigration break junctions, cross-bar junctions, and nanopore junctions) experimentalists are now able to form stable molecular bridges and explore the functionalities by examining the dynamic response of the electrical characteristics to the external fields.

An essential ingredient for conducting reliable single-molecule device characteristics has been a technique that can identify molecular signatures of the molecule in the junction. In the early discussions of quantized conductance of metal quantum point contacts, high resolution transmission electron microscopy (TEM) has played an important role by elucidating the evolution of metallic single-atom chains by *in situ* observations of thermally-driven shrinkage of a nano-junction; and meanwhile simultaneous measurements of the conductance traces provided solid evidence of conductance quantization in the Au single-atom contacts [12,13]. Applications of the TEM approach to single-molecule junction characterizations will no doubt revolutionize the field of single-molecule electronics by providing valuable information *in situ* concerning molecular conformations and metal-molecule contact configurations at the atomic level. Unfortunately, however, organic molecules can be easily burned out by heat generated upon irradiating high-energy electron beam. Therefore, TEM observations of molecular junctions have not been realized (except for fullerenes possessing high temperature stability [89–91]).

#### 2.4.1. Raman Spectroscopy

One of the practical methods for identifying molecular junctions is to incorporate Raman spectroscopy, a well-established technique in chemistry for fingerprinting molecules [92–94]. Particularly, the structure of single-molecule bridges, a molecule residing in a nanospace between two electrodes, can benefit from the photo-excited surface plasmons on the metal surface that augments local fields there and leads to orders of magnitude enhancements of Raman signals. This surface enhanced Raman spectroscopy has been applied on a molecular bridge formed by the electromigration and mechanical break junction methods [92–94]. It was demonstrated that electromagnetic field enhancement necessary for single-molecule detections can be achieved by using Au electrodes. Especially, combined measurements of Raman spectrum and single-molecule conductance were found to provide a wealth of information that enables us to correlate the structural variations and the conductance states of a single-molecule bridge [94]. Furthermore, a unique outcome of this approach is that it can be used to explore inelastic electron-phonon interactions and concomitant local heat dissipation in current-carrying molecular junctions by analyzing the anti-Stokes Raman emission [93,95,96]. Although quantitative assessment of the junction effective temperature is still elusive and depends on models, the bias dependence of the Stokes/anti-Stokes peak intensities can reveal relative contributions of specific molecular vibrational modes to the local heating and cooling processes [95,96].

#### 2.4.2. Inelastic Electron Tunneling Spectroscopy

**Figure 5.**
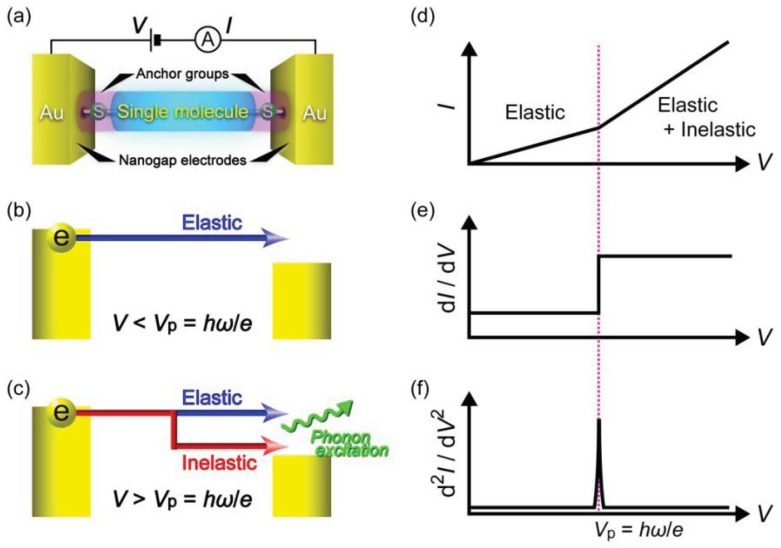
Inelastic electron tunneling spectroscopy for single-molecule fingerprinting. (**a,b**) Electrons tunnel through the molecule elastically at the low voltage; (**c**) When *V* exceeds *V*_p_, a few per cent of the tunneling electrons couple strongly with a certain vibrational mode with energy *hω*, where *ω* is the molecular vibration frequency; (**d**) Current *I* flowing through a single-molecule tunneling junction increases linearly with the bias voltage *V* in the elastic tunneling regime; (**e**) At *V* > *V*_p_, the inelastic channel opens and the differential conductance increases discretely (**f**) causing peaks in the d^2^*I*/d*V*^2^-*V* vibrational spectrum.

Another useful single-molecule technique is inelastic electron tunneling spectroscopy (Figure 5(a)) [97–100]. At low voltages, electrons elastically tunnel through a molecule with a relatively large HOMO-LUMO gap. When the voltage *V*_b_ exceeds *ħω/e*, a few percent of the total tunneling electrons impart the kinetic energy to excite molecular vibrations, where *ω* and *e* are the frequency of a molecular vibration mode and the elemental charge, respectively [100]. The inelastic electron tunneling contributes positively to the total current in case when *T* < 0.5 and negatively for *T* > 0.5, where *T* is the transmission probability in the conductance channel [101,102]. Thus, the onset of IETS-active molecular vibration mode excitations originates upward (downward) peaks in the ${\text{d}}^{2}I/\text{d}V_{\text{b}}^{2}-V_{\text{b}}$ curves for tunneling (ballistic) molecular junctions (Figure 5(b)) [101,102]. As electron-vibration interactions exist in virtually any types of molecules, the vibrational spectra can be used as a useful molecular signature for single-molecule identifications with high specificity. Nevertheless, unlike Raman spectroscopy, selection rules for the coupling of tunneling electrons with the molecular vibration motions are not established [103]. Therefore, the vibrational spectroscopy accompanies theoretical calculations of molecular vibration modes that couple strongly to electrons.

IETS has been used as a tool for studying the conformation and adsorption sites of molecules adsorbed on a metal substrate with a vacuum gap at the tip side using STM [97–99,104]. Recently, it was also applied to metal/molecule/metal junctions [105–110]. The vibrational spectra were often not symmetric about zero bias and slightly differed junction by junction [111,112]. The asymmetry and variation are attributed to the random nature of molecular orientations and metal-molecule contact configurations formed in break junction experiments and the different coupling strength at the right and left sides of the molecule [113]. Averaging more than one spectrum gives peak voltages that could often be assigned to the Raman-active vibrational modes. The validity of the single-molecule vibrational spectroscopy could be verified by demonstrating the thermal and modulation broadening of the spectrum peaks [112,114–116]. Nowadays many break junction experiments use IETS to check for the presence of molecules in the electrode gap. Recently, IETS measurements were reported on Au/single-benzenedithiol/Au junctions [117,118]. Despite that benzenedithiol is the most studied π-conjugated molecule, the single-molecule conductance has been elusive and spans more than three orders of magnitude [117]. The expansive distribution was explained theoretically by the large impact of the molecular orientations and the bonding sites on the electron transmission probability [37,39]. Kim *et al.* have conducted systematic measurements of vibrational spectra at the various conductance states extending from 0.5 *G*_0_ down to 10^−3^*G*_0_ [117]. The results showed clear evidence that these conductance states originates mainly from electron transport through single conductance channel through a benzenedithiol molecule connected to two Au electrodes: vibrational spectra with dips and peaks corresponding to the IETS-active vibrational modes at the junction conductance above and below 0.5 *G*_0_, respectively [117].

Beyond single-molecule fingerprinting, when combined with mechanical break junction measurements, IETS has been proved to be useful for studying the correlation between the molecular conformation and the electron transport properties [119,120]. In these interesting experiments, alkanedithiol single-molecule junctions were stretched little by little by several Ångstrom using micro-fabricated MCBJs and measured the vibrational spectra at different elongation states. It was possible to identify a mechanical transition from *trans* to *gauche* conformations [119,120]. Furthermore, the Au-S stretching mode changed appreciably when the single-molecule conductance jumped from the high- to the low-conductance states, manifesting that the conductance drop was originated from mechanical deformation of the metal-molecule contact configurations [119,120]. This new application of IETS analysis may provide a way of designing the mechanically-controlled single-molecule switches.

The vibrational spectroscopy can also be implemented in a single electron tunneling regime by using the electronic gating effects. In this method, the differential conductance of a single-molecule junction is measured using a three-terminal device and plotted as a function of the source-drain voltage and the gate voltage. The thus obtained stability diagram reveals fine structures assigned to the excitation energies of the Raman- and IR-active molecular vibrational modes of the single-molecule bridges, thereby constituting another useful strategy for single-molecule fingerprinting [3,121].

#### 2.4.3. Inelastic Noise Spectroscopy

In single molecule experiments, the stochastic nature of molecular fluctuations in general acts as an inevitable source of noise that obscures fine details and complicates the identification of the molecular identity. In contrast, it was reported that molecular-vibration-induced current noise can itself be a useful molecular signature for single-molecule identifications [122]. In this study, *I-V*_b_ characteristics of Au/hexanedithiol/Au single-molecule junctions were measured. The electric current noise in a single-molecule hexanedithiol sandwiched between a pair of electrodes defined as the standard deviation of current fluctuations at a constant voltage increases in a stepwise linear fashion synchronous to excitations of its distinct vibrational modes active in the electron-phonon interaction, which thereby enable single molecule fingerprinting through examining the noise spectra [122]. This unambiguously suggested that the voltage-dependent electric noise stems from inelastic interaction between electrons and molecular vibrations. Local ionic and electron heating in the current-carrying single-molecule junctions raises the effective temperature and would enlarge the Johnson-Nyquist noise [68,123]. Therefore, it was expected theoretically that information relevant to local heating can be extracted from the voltage-dependent noise in the molecular junctions. However, the high-bias noise in the hexanedithiol single-molecule bridges was too large to be attributed to local heating effects alone. It may be necessary to analyze the frequency dependence of the noise spectra in order to deduce the certain extent of local heating contributions embedded in the experimentally measured electric noise. As such, the inelastic noise spectroscopy will not only be a complementary technique to IETS but also has a potential to be used as a unique probe for investigating local heating in single-molecule junctions [124,125].

## 3. Single-Molecule Electronics Devices

As we have seen in the preceding section, the progress in single-molecule junction fabrication technologies and statistical approaches for evaluating the electrical properties has led to better understanding of electron transport in an individual molecule sandwiched between metal electrodes. Besides the academic interest, industries demand for discoveries of useful functions of molecules for various device applications. In this section, we review recent efforts for development of functional single-molecule bridges that can act as electronic components such as switches, diodes, and transistors.

### 3.1. Single-Molecule Switches

A conductor capable of changing its impedance between two distinct levels controllably and repeatedly can be used as a switching device for storing information as we do so with flash memories. The nanometer size of single-molecule switches promises applications for memory devices by realizing unprecedented integration densities. For this, it is important to demonstrate high speed conductance switching with a long retention period and good endurance by single-molecule junctions under ambient conditions.

Binary switching of single-molecule conductance was first inferred in scanning tunneling microscopy studies of individual molecules adsorbed on a surface [126,127]. Isolated alkanedithiol molecules embedded in alkanethiol self-assembled monolayer on a Au (111) surface were imaged as bright spots. The single-molecule images revealed blinking of the bright dots in the consecutive images of the same area. This phenomenon was attributed to on-off of the conductance states of the alkanedithiols associated with breaking and formation of the Au-S links. The blinking frequency was found to increase at high temperatures, suggesting thermally activated bond breaking processes at the metal-molecule contacts [127]. In break junction experiments, the two-level stochastic switching has also been observed in various metal/molecule/metal junctions, which were attributed to thermal fluctuations of the electrode-molecule bonding sites and concomitant change in the electrode-molecule coupling strength [128–130]. These findings indicate that binary switches can be made by exploiting the geometrical dependence of the single-molecule conductance through developing a way to control the molecular conformations and metal-molecule contact configurations [131].

#### 3.1.1. Electric Field Control of Molecular Conformations

**Figure 6.**
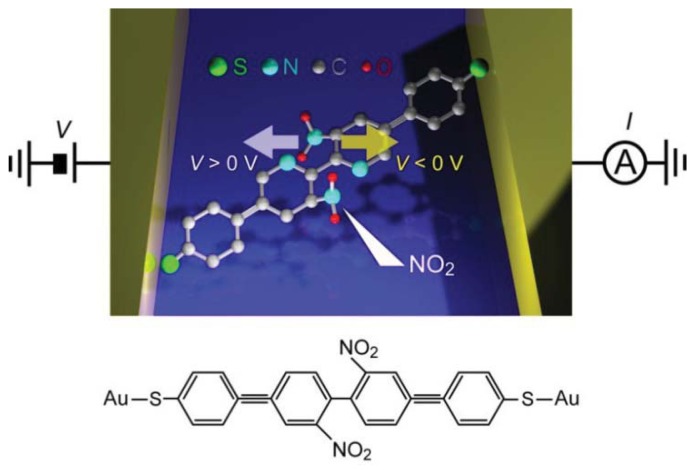
Bias voltage controlled bipolar binary conductance switching in single-molecule bipyridyl-dinitro oligophenylene-ethynylene dithiol sandwiched between Au nanoelectrodes. The conductance jumps to the on state at 0.8 V during sweeping the bias in the positive direction, which was attributed to conformational change of the dithiol molecule by the electrostatic forces originated from the electric field across the electrode gap at the dipole moments induced by the NO_2_ groups (white arrow). Subsequently, the junction conductance was switched back to the off state by decreasing the bias voltage to −1.0 V associated with the electrostatic force now acting in the opposite direction (yellow arrow) that reverses the conformational change. The on-off switching was reproduced in the successive voltage cycles.

Controlled conductance switching has been demonstrated for various kinds of molecules using the electric field tuning of molecular conformations [132–137]. Lörchester *et al.* [137] developed two-terminal single-molecule switches consisting of bipyridyl-dinitro oligophenylene-ethynylene dithiols (BPDN-DT) connected to two Au electrodes (Figure 6).

*I-V* curves of BPDN-DT single-molecule junctions measured using a micro-fabricated MCBJ showed a jump from the low- to the high-conductance states at about 1 V during positive voltage sweep from 0 V to 1.5 V. Subsequently sweeping back to 0 V, the junction did not switch off and showed a hysteresis. However, negative sweep from 0 V to −1.5 V reset the switch to the off-state at about −1 V. Thereafter, the on-off switching was reproducible under repeated sweeps for more than 500 cycles from −1.5 V to 1.5 V. Stochastic switching was not observed because the measurements were performed at low temperatures (100 K) where thermal fluctuations were suppressed. The underlying mechanism of the bias-induced binary switching was explored by performing control measurements on bipyridyl oligophenylene-ethynylene dithiols (BP-DTs). The BP-DT junctions possessed higher conductance than that of BPDN-DT, which was attributed to the absence of NO_2_ units in the pyridine rings that leads to a twisted conformation by the steric repulsion and accompanying decrease in the *π*-overlap (Figure 6). Interestingly, BP-DTs showed neither the switching characteristics nor hysteresis in the *I-V* curves. This manifested a predominant role of the dressed NO_2_ groups of BPDN-DTs on the bistable switching [137]. Theoretical calculations suggested molecular rotation by the electrostatic force acting on the NO_2_-induced dipoles during voltage sweep [138].

The conductance states of BPDN-DT junctions could also be controlled by applying a voltage pulse: a positive pulse (+1.6 V) could bring it to the on-state, while a negative pulse (−1.6 V) switched it back to the off-state. Using the voltage pulses, the on/off conductance ratio at the read voltage of 1.1 V was estimated to range from 7 to 70. Furthermore, the retention period was longer than several minutes at 100 K. However, the excellent performance of BPDN-DT switches could not be realized at room temperatures as the junction collapsed during voltage sweep [137] presumably due to the local heating and/or the field-induced migration of Au atoms at the high voltages [67–70]. This high-field instability has been recognized as a common issue for potential electronic device applications of single-molecule junctions.

#### 3.1.2. Mechanical Control of Metal-Molecule Contact Configurations

**Figure 7.**
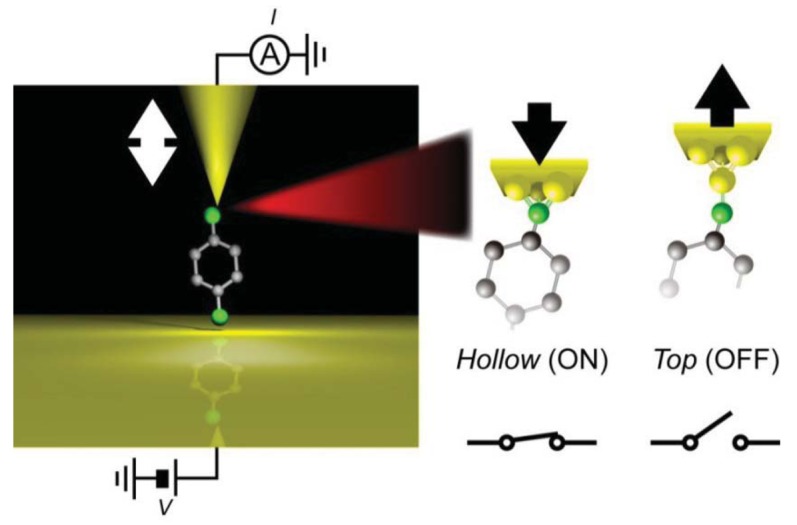
Mechanically-controlled conductance switching of single-molecule junctions. Binary conductance switching occurs synchronous to the sinusoidal modulation of the tip motion that repeatedly deforms metal-molecule contact structure between two stable configurations such as hollow-to-top Au-thiol bonding sites.

Alternatively, mechanical forces can be used to manipulate the molecular junction geometries. The idea is based on deformation of bistable electrode-molecule contact configurations by pulling and compressing a single molecule junction (Figure 7) [131].

Recently, Quek *et al.* reported mechanically-controlled conductance switching in Au/4,4′-bipyridine (BPD)/Au single-molecule bridges [139]. They repeatedly formed molecular junctions by moving the tip of a SPM in and out of contact with the Au substrate in a dilute solution of BPDs. A histogram constructed with thousands of conductance traces measured during junction stretching revealed two distinct single-molecule conductance states. The BPD junctions could switch between the two states with a several-angstrom sinusoidal motion of the tip for several on/off cycles. Mechanical deformation processes responsible for the binary switching were further investigated by the first principle calculations on the geometrical dependence of the electron transmission through the single molecule junctions, which suggested compression-induced jumping of a BPD molecule between the two binding sites on the Au tip surface [139].

Another example of a bistable mechanical switch was reported in Au/hexanedithiol/Au systems [120]. In this system, multiple single-molecule conductance states have been reported by several groups. To demonstrate mechanically-controlled conductance switching, stable hexanedithiol single-molecule junctions were formed at room temperatures in a vacuum by using a micro-fabricated MCBJ. By adding a 3 Å sinusoidal displacement of the nanoelectrode positions, the junction conductance could be switched back and forth between 1.3 m*G*_0_ and 0.4 m*G*_0_ at a frequency up to 0.1 kHz. The switching mechanism was explored by the independent IETS measurements carried out at low temperatures. The increase in the relative intensities of vibrational peaks for Au-S and CH_2_ stretching modes together with decrease in the CH_2_ wag mode feature observed upon the mechanical transition from the high- to the low-conductance states indicated a change in the Au-S motif from hollow-hollow to top-top arrangements (Figure 7) [120].

The advantage of the mechanical approach is that it does not require high voltage for controlling the on-off, unlike the case for operating BPDN-DT switches [137], and therefore does not suffer from the high-bias instability issues. For high-density integration of the mechanically-controlled single-molecule switches, it is essential to replace the SPM break junction or the MCBJ apparatus with other technologies. One possible way is to implement electromechanical tuning of the electrode gap by the electrostatic field imposed through the air gap using a back-gate device configuration [140], while forming single-molecule bridges with the electromigration break junction method.

#### 3.1.3. Photochromic Switches

**Figure 8.**
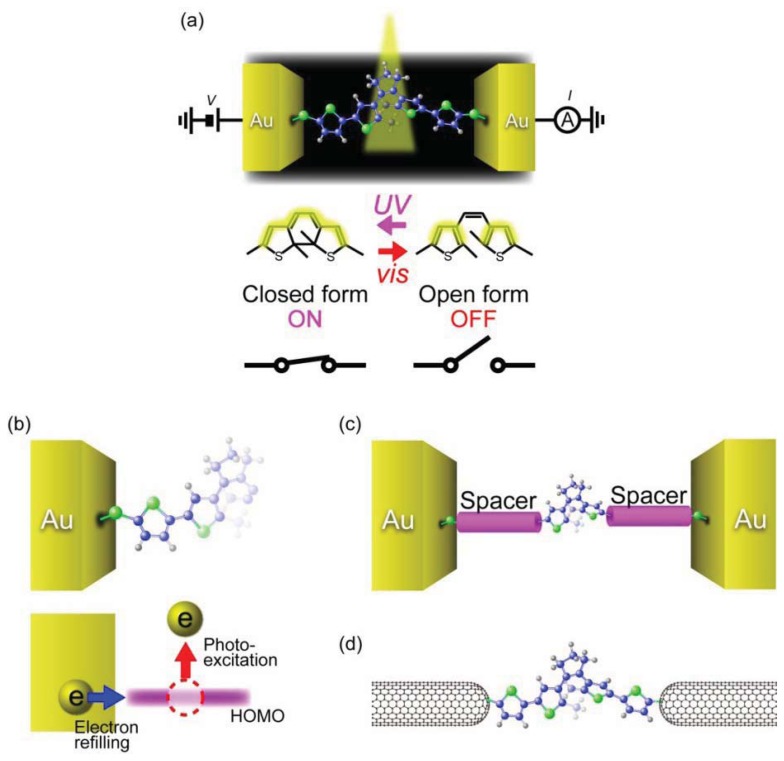
Photochromic single-molecule switches. (**a**) The ring opening and closing photochromic reactions of diarylethene by visible and UV light irradiation can be used for photo-controlled switching between the high- and the low-conductance states of the closed and the open forms; (**b**) In experiments, however, only one-way conductance switching has been observed for dithienylethenes bridged between Au electrodes due to quenching of the closing reaction by the refilling of the electrons by virtue of the feasible charge transfer between Au and the HOMO; (**c**) Reversible photo-switching of diarylethene single-molecule junctions was accomplished by inserting spacer molecules to reduce the Au-molecule interactions or (**d**) by replacing the Au electrodes with carbon nanotubes.

It has also been demonstrated that conductance of a single-molecule junction can be switched by light control of the reversible photochromic reactions (Figure 8(a)) [141]. 1,2-Diarylethene is a photochromic molecule that undergoes reversible ring opening and closing processes upon irradiating with visible and UV light, respectively [142]. Unlike azobenzene that only changes its length by *trans-cis* isomerization, diarylethene molecules can drastically change their electrical characteristics through transforming from the non-conjugated open form to the conjugated closed form [143]. This electronic structure difference was theoretically predicted to provide a large conductance ratio of 10–100 between the open and closed isomers when sandwiched between electrodes [144]. Furthermore, the experimental observations that the phtochromic reaction of diarylethenes proceeds within picoseconds in a solution suggested THz-level photo-switching speed [145,146]. These unique characteristics of diarylethenes are promising for ultra-fast single-molecule photo-switch applications.

Single-molecule conductance of diarylethene derivatives has been measured using break junction techniques [147–150]. Dulíc *et al.* [147] examined light-induced conductance switching of dithienylethenes with thiophene rings connected at both sides and a dithiol anchoring group. They formed single-molecule junctions at room temperatures using lithographically-defined MCBJs. The dithienylethenes were initially in the closed form wherein the density of states is delocalized through the entire molecule. The *I-V*s obtained in dark environment showed non-linear characteristics with a low-bias resistance of 1 MΩ. Visible light was then irradiated on the junction and the response of the conductance was monitored. This led to a three orders of magnitude drop of the conductance, which was interpreted as stemming from the ring opening reaction in the dithienylethene unit. Despite the excellent on/off conductance ratio, the switching speed was found to be unexpectedly slow: the on-to-off switching occurred about 10 s to 6 minutes after the visible light irradiation; and off-to-on switching could not be even observed upon UV illumination exposure [147]. The one-way switching properties were attributed to quenching of the photo-excited closing reaction by the fact that the HOMO level of the open isomer overlaps with the high density of states of the Au electrode, which facilitates fast electron transfer to the molecule and eventually shorten the lifetime of the hole (Figure 8(b)) [146,147].

Attempts have been made to realize reversible photochromic switching by weakening the interaction between the HOMO of the open form diarylethene and Au electrodes (Figure 8(c)). For this, thiophene spacer at both sides of dithienylethene was replaced by phenyl rings [148–150]. Although the on/off ratio decreased by several factors, the phenylene spacer enabled the molecule to switch back to the closed form by UV irradiation [149,150]. Reversible photochromic switching was also attained by using semiconducting single-walled carbon nanotubes as electrodes (Figure 8(d)) [151]. These results may guide the molecular design for further improvements of the switching speed of diarylethene-based photo-switches.

### 3.2. Single-Molecule Diodes

**Figure 9.**
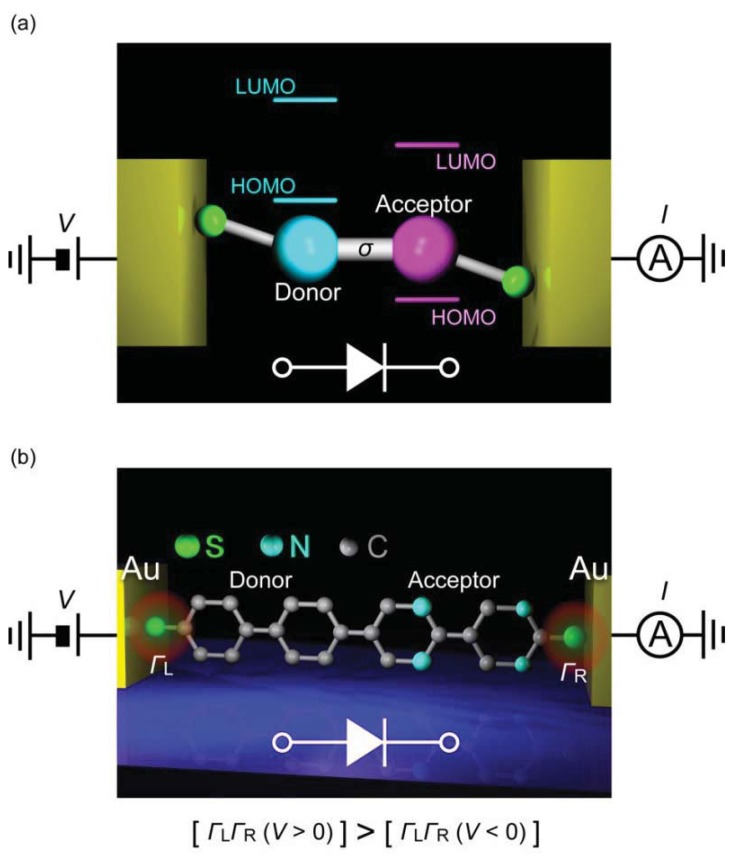
Single-molecule diodes. (**a**) Schematic model of the Aviram-Ratner molecular diode comprised of a donor-*σ*-acceptor (D-*σ*-A) structure. The molecular orbitals are localized on the donor and the acceptor units by virtue of the insulating barrier at the *σ* bond. Because of the asymmetric structure, the HOMOs and LUMOs of the D-*σ*-A system align with the electrode Fermi level more easily in one bias direction than the other, thereby causing current rectification; (**b**) A schematic illustration of the dipyrimidinyl-diphenyldithiol single-molecule junction with a donor-acceptor molecular architecture. A diode-like behavior was observed wherein the electron flow from the left to the right of the junction was suppressed. The rectification characteristics were explained by the voltage-induced change in the molecular orbital phase and amplitudes that cause asymmetric metal-molecule contact coupling *Γ*_L,R_ at the pyrimidinyl and phenyl ends.

A diode is a two-terminal electronic component that facilitates current flow in one (forward) bias direction and suppresses under the opposite (reverse) direction. Molecular design for diodes is essentially similar to the p-n junction configuration and consists of donor and acceptor molecules connected via *σ*bond (Figure 9(a)) [1]. For the donor (D) and the acceptor (A), electron donating and withdrawing substituents are often used to chemically engineer the frontier molecular orbital levels of *π*-conjugated units, while the *σ* link serves as an insulating barrier for electrically separating the two segments. Original device concept exploits the energetically facile acceptor-to-donor electron transfer in D-*σ*-A systems for current rectifications [1]. Nevertheless, asymmetric *I-V* characteristics can arise from many factors. In the early experiments on Langmuir-Blodgett films of C_6_H_13_Q-3CNQ, both positive and negative rectifications were observed [152–155]. The variation was explained by the electrostatic shifts of the molecular orbital levels that could cause rectification characteristics opposite to the Aviram-Ratner mechanism depending on the conditions for resonance between the electrode Fermi level and HOMO or LUMO [156,157]. Using mechanical break junction techniques, it was also found that difference in the electrode-molecule coupling at the both sides can give asymmetric *I-V* behavior even for essentially symmetric molecules [158,159]. These issues complicate the interpretation of the experimentally observed rectification characteristics of molecular junctions.

The first single-molecule diodes have been fabricated using a lithographically defined MCBJ [160]. The molecules had a donor-acceptor structure with one of the phenyl group being functionalized with four fluorine atoms. The electronic states of the two functional units were localized by reducing the overlap of their *π*-orbitals using the large torsion by steric hindrance of methylenes at the D-A link. Molecular junctions were formed using dithiol anchoring groups with Au MCBJ electrodes. Rectification behavior was observed with the current ratio of up to 10. The *I-V* asymmetry was due to the different heights of the conductance steps at the positive and negative biases that originated from crossing of the occupied and the unoccupied molecular orbital levels by virtue of their electrostatic shifts under the applied bias. Contributions of metal-molecule contacts were also found to be negligible by measuring D-D and A-A molecules as controls that gave symmetric *IV*s. Although density functional theory calculations nicely explained the electron transport characteristics, the random nature of the molecular orientations in the MCBJ approach led to junction-to-junction variation in the *I-V* curves and posed a difficulty to clarify the actual bias direction along the D-A molecule [160]. Recent SPM break junction experiments by Díez-Pérez *et al.* [161] incorporated two different protection groups to control the orientations of diode molecules. They formed a self-assembled monolayer of dipyrimidinyl-diphenyl molecules on a Au (111) substrate by deprotecting the thiol at the diphenyl sides. Removing the protecting group at the dipyrimidinyl side allowed formation of molecular junctions by approaching a Au tip to the substrate via S-Au bonds. This excellent procedure regulated the orientation of molecules to align vertically with the dipyrimidinyl unit heading upward, whereby enabling unambiguous definition of the bias direction. The single-molecule conductance was determined using the repeated break junction method, which was then used as a reference for fabricating single-molecule junctions, ahereas symmetric *IV*s were acquired for tetraphenyl molecules, the dipyrimidinyl-diphenyl molecules clearly demonstrated rectification characteristics with the average current ratio of 5.

The fact that the current was suppressed in the negative bias regime suggested an important role of asymmetric electrode-molecule coupling that yields lower threshold voltage in the positive bias regime for attaining resonance between the anode and the molecular HOMO (Figure 9(b)) [162]. It has now become technically available to form single-molecule diodes with well-defined molecular orientations. The low rectification ratio is the remaining issue, which requires improved design of D-*σ*-A architecture. One way may be to pursue the molecular level structure with small energy gap between the donor HOMO and the acceptor LUMO so as to utilize the delocalized resonance states under electrostatic crossing of these two levels.

### 3.3. Single-Molecule Field-Effect Transistors

Most of today's integrated circuit chips are based on metal-oxide-semiconductor field effect transistors (MOSFETs). As the gate length is already far below 100 nm and continues to become smaller, the solid-state silicon devices are starting to face fundamental physical limits, e.g., dopant fluctuations, short channel effects, and oxide-gate tunneling. Whether single-molecule junctions can find applications as FETs is of practical interest for further downscaling of transistors to sub-10 nm scale.

Theoretical verifications of back-gated single-molecule transistors have predicted that the conductance can be modulated by the gate-field induced electrostatic shifts of the molecular energy levels with respect to the electrode Fermi level [163,164]. However, screening by the charge rearrangements in the electrode is suggested to weaken the gate effect considerably for the nanometer scale gate length of molecular junctions [165]. A challenge to form single-molecule FETs has thus been to put a gate electrode at the vicinity of the molecule.

Development of single-molecule FETs has been vigorously explored in the past decades. Three-terminal molecular junctions were fabricated often by utilizing electromigration break junction method to form source and drain metallic electrodes with nanometer separation on a bottom gate electrode using a silicon micro-fabrication technology [3,77]. To minimize the gate-to-molecule distance and thereby overcoming the screening effects, Al was frequently employed for the gate electrode material wherein ultra-thin Al_2_O_3_ natural oxide layer served as the gate dielectric. Gate modulation of single-molecule conductance has been demonstrated for various types of *π*-conjugated molecules [3–5,166–169]. These experiments observed staircase-like structures in the *I-V* curves. The differential conductance plotted against the source-drain and gate voltage revealed diamond shaped profiles that manifests Coulomb blockade in a weakly coupled molecule to the leads. Although interesting phenomena such as single electron tunneling and Kondo effects have been found in this weak coupling regime, demonstration of the original concept of single-molecule FETs, *i.e.*, continuous modulation of the single molecule conductance by electrostatic modification of the energy gap between the frontier levels and the electrode Fermi level, required fabrication of molecular junctions with stronger electrode-molecule coupling.

The role of electrode-molecule coupling strength on the gate modulation of single-molecule electron transport has been investigated by Danilov *et al.* [170]. In this ingenious work, electrode-molecule coupling was tailored by incorporating CH_2_ units at both sides of the oligophenylene-vinylene that served as tunneling barrier for localizing the electronic states on the molecule and weaken the electronic coupling to Au leads. By forming three-terminal single-molecule junctions using the mask-defined nanogap device with an Al_2_O_3_/Al bottom-gate configuration, they found non-linear *I-V* curves with no conductance gap at low voltages for oligophenylene-vinylene molecules anchored directly to the Au electrodes via S-Au bonds, suggesting coherent tunneling of electrons through the molecule. For these molecules, the differential conductance changed little with the gate voltage. The absence of Coulomb blockade signatures indicated strong coupling at the electrode-molecule contacts that smeared out the single electron charging effects. On the other hand, the junction conductance decreased by orders of magnitude for the same molecules with CH_2_ barriers inserted. Current flowing through the molecules could be modified appreciably by the gate voltage and revealed Coulomb diamond in the differential conductance diagram. These results directly showed the critical role of electrode-molecule coupling on the electron transport characteristics [168]. Unfortunately, however, large gate modulation of the conductance was not achieved, presumably because of the weak gate coupling by screening of the electrostatic field due to the proximity of the electrodes.

**Figure 10.**
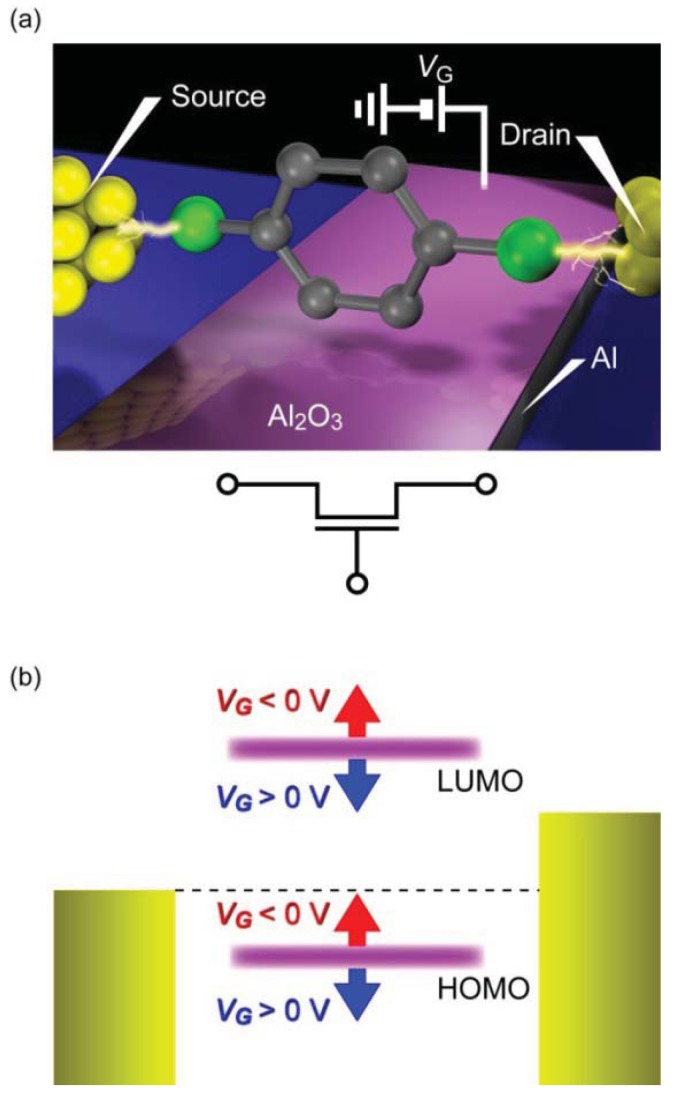
Au/1,4-benzenedithiol/Au single-molecule FETs. (**a**) A schematic view of the single-molecule FET. The Al/Al_2_O_3_ bottom gate electrode was used for modulating the source-drain current flowing through an individual benzenedithiol molecule; (**b**) The HOMO and the LUMO are fixed at the positions relative to the Fermi level (dotted line) with the source-gate voltage *V*_G_. The orbital energy levels can be shifted upward (downward) by the negative (positive) *V*_G_.

Recently, the first proof-of-principle demonstration of single-molecule FETs has been reported by Song *et al.* [171]. In this excellent experiment, benzenedithiol molecules were bridged between electrode nanogap formed using the electromigration break junction method, and a back gate device configuration with conventional Al_2_O_3_/Al electrode was used to investigate the electrostatic modulation of current flowing through the molecule (Figure 10(a)). Au/single-benezenedithiol/Au structure is one of the most widely studied *π*-conjugated molecules with electronic states delocalized over the entire junction [22,117]. The electron transport is coherent tunneling as suggested by the gap-less non-linear *I-V* characteristics with the *G*_SMC_ spanning vastly from 0.1 *G*_0_ to 0.0001 *G*_0_ depending on the molecular conformations and the metal-molecule motifs [117]. Although the yield was not so high, less than 10%, the devices with low-bias conductance of 0.01 *G*_0_ that corresponds to typical single-molecule conductance of Au-benzenedithiol-Au junction [22] exhibited a large modulation of the *I-V* curves with the gate voltage. Transition voltage spectroscopy was performed to investigate the gate modulation of the energy gap between the electrode Fermi energy and the nearest molecular level [166–168]. In this method, voltage where a minimum in (ln*I*/*V*^2^) *versus* 1/*V* plots occurs is deduced. This transition voltage can be utilized as a qualitative measure of the charge injection barrier height that corresponds to the energy level whereat the broadened resonance level contributes to the conductance at a certain extent [172–175] (it should also be noted however that care is needed when assessing molecular orbital levels from the transition voltage unless the electrostatic potential profile along the current-carrying molecular junction is uncertain, as is the case in most of the experiments [174,176,177].).

The positive linear scaling of the transition voltage with the gate voltage was an indication of electron transport through the HOMO level [171]. The gate coupling strength, which was about 0.2 eV/V, could also be estimated from the slope. In addition to this, IETS analyses were applied to the molecular junctions. The vibrational spectra showed pronounced peaks at well-defined voltages corresponding to the IETS-active vibration modes of Au/benzenedithiol/Au systems. Furthermore, it was found that the peak height became significantly enhanced with increasing the gate voltage. These findings not only served to validate the presence of current-carrying benzenedithiol in the electrode gap but also inferred strong coupling between the HOMO and the molecular vibrational modes at near resonance [171,178]. Overall results supported gate modulation of the single-molecule conductance via the electrostatic tuning of the molecular orbital levels (Figure 10(b)). Although single-molecule FETs have proven to work as predicted theoretically, the low device yield is a critical issue. Technical barriers to be overcome include formation of well-defined metal-molecule contacts with strong coupling, controls of gate-to-molecule distance, and mitigating the electrode screening. Perhaps, these challenges can be overcome in part by exploiting graphene as source and drain electrodes [88,179]: the atomic-sheet geometry provides sub-nanometer gaps between the molecular bridges to the bottom gate and also reduces the screening effects.

## 4. Thermoelectric Transport in Single-Molecule Junctions

**Figure 11.**
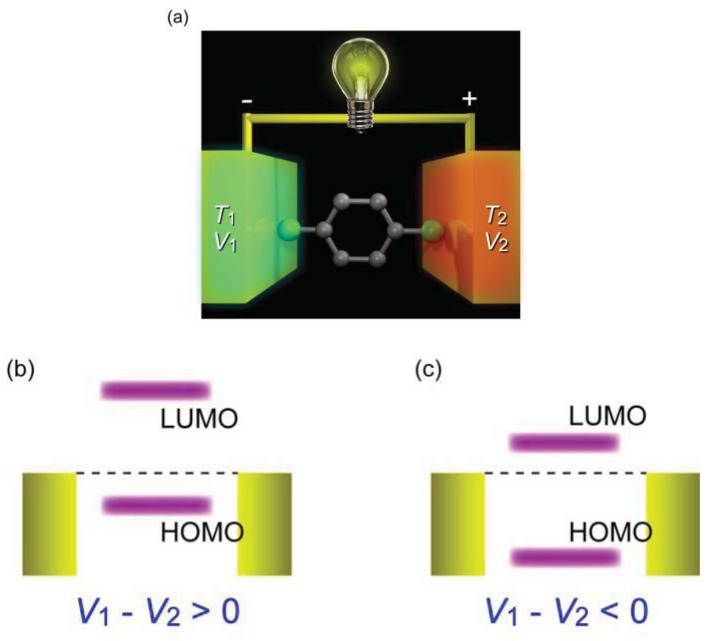
Thermopower measurements of Au/benzenedithiol/Au junctions. In experiments, molecular junctions were formed repeatedly by moving the tip in and out of contact with the self-assembled monolayer on the Au substrate. The substrate was heated to *T*_2_ higher than the ambient *T*_1_ up to several tens of Kelvins. Current was measured during tip approach processes using the current amplifier until the conductance reached at the set point. Subsequently, the circuit is switched and the voltage was measured using the voltage amplifier while stretching the molecular junction. (**a**) The thermoelectric voltage Δ*V* = *V*_2_−*V*_1_ could be measured during molecular junction stretching processes under the tip-substrate temperature difference Δ*T* = *T*_2_−*T*_1_. This indicates thermoelectric energy conversion by the molecules connected to two electrodes. The junction thermopower can be deduced by measuring Δ*V*/Δ*T*; (**b,c**) The sign of the thermoelectric voltage can be used as a diagnostic tool for elucidating the Fermi alignment of the metal-molecule-metal junctions: HOMO (LUMO) is closer to the electrode Fermi level when the thermoelectric voltage *V*_1_ − *V*_2_ is positive (negative).

In addition to the strong motivation for the development of molecular electronics devices, there is a growing interest in understanding thermoelectric transport in single-molecule junctions [72]. When one side of a material is heated to *T*_1_ while the other is kept at *T*_2_, free charges tend to accumulate at the cold end and thereby induce a potential difference Δ*V* (Figure 11(a)). This thermoelectricity enables direct conversion of heat energy to electricity, and vice versa, without any use of mechanical components such as compressors or turbines. Together with the vast scalability of the thermoelectric devices, molecular junctions can be a promising candidate for energy harvesting in mobile electronics being a light weight and flexible planar thermoelectric module. To make this real, it is essential to develop *p* and *n* type molecular wires with high thermoelectric properties.

### 4.1. Thermopower of Molecular Junctions

Thermoelectric energy conversion efficiency is represented by the material's dimensionless figure of merit *ZT* = *σS*^2^*T*/*κ*, where *σ* is the electrical conductivity, *S* = −Δ*V*/Δ*T* is the thermopower (or Seebeck coefficient), and *κ* is the thermal conductivity. It has long been pursued to develop thermoelectric materials with *ZT* > 3. However, achieving enhancement of *σ* and decrease in *κ* at the same time is intrinsically difficult in bulk compounds because heat is carried not only by photons but also by charge carriers so that a rise in the electrical conductivity is counteracted by the concomitant increase in the electronic contribution of thermal conductivity as stated in the Wiedemann Franz law. This has been a critical barrier for attaining *ZT* > 1 despite the fact that (Bi,Sb)Te alloys were found to possess*ZT* ∼ 1 already in 1950s [180].

Recently, an increasing amount of research is focusing on utilizing low-dimensional nanostructures for improving *ZT* of bulk materials [181,182]. Quantum well superlattices have proven to be a promising system that allows independent control of the thermal conductivity through the increased phonon scattering rates at the interfaces [181,183]. By this effect, unprecedented *ZT* > 2 has been reported for Be_2_Te_3_/Sb_2_Te_3_ [184]. Another potential strategy is to exploit charge confinements in a quantum dot, which makes use of a delta-function distribution of the density of states at the discrete energy levels to obtain high thermopower [185,186]. As single-molecule junctions share essentially the same feature, Lorentzian distribution of density of states at HOMO and LUMO levels defined by the molecule-electrode coupling strength, they may also be potentially important thermoelectric materials [187,188].

Advances in theoretical modeling of the conductance and the thermopower of a single-molecule junction offer a physical picture for how to improve the power factor *σS*^2^ [72,189–192]. As thermoelectric generator operates under zero bias voltage, *G* in the expression of *ZT* corresponds to the electron transmission of a single-molecule junction at the Fermi level *E*_F_, which can be described as:

(2) $$G=\left(2e^{2}/h\right)T\left(E\right){|}_{E\text{F}}$$

The transmission function is determined by the frontier molecular orbital levels (*E*_HOMO_ and *E*_LUMO_) and the metal-molecule coupling at the left (*Γ*_left_) and right side (*Γ*_right_) of the junction that broadens the HOMO and LUMO energies. Analytical expression of *T*(*E*) is derived as:

(3) $$T\left(E\right)=\sum \left(\Gamma_{\text{left}}\Gamma_{\text{right}}\right)/\left[\left(E-E_{\text{i}}\right)+{\left(\Gamma_{\text{left}\;+}\;\Gamma_{\text{right}}\right)}^{2}/4\right]$$

from single-particle scattering theory, where summation is over *E*_i_ = *E*_HOMO_ and *E*_LUMO_ [189]. This predicts that *G* takes a maximum at the resonance when the nearest molecular orbital level aligns with the electrode Fermi level that makes *T*(*E*) ∼ 1 for a symmetric junction with *Γ*_left_ ∼ *Γ*_right_. Similar to the conductance, thermopower is determined by the line shape of the transmission curve at the Fermi level *E*_F_. The simplified Landauer expression within a single-particle picture of Seebeck coefficient of single-molecule junctions under assumptions of low temperatures and a far-from-resonance condition is written as:

(4) $$S=-\left(\pi^{2}k_{\text{B}}^{2}T_{0}/3eT\left(E\right)\right)\left(\partial T\left(E\right)/\partial E\right){|}_{E\text{F}}$$

where *k*_B_ and *T*_0_ are the Boltzmann constant and the temperature of the junction, respectively [189]. It is anticipated from the Lorentzian-shaped transmission curves peaked at the HOMO and LUMO energy levels that *∂T*(*E*)/*∂E* attains a maximum and changes its sign at near the resonance states. These theoretical aspects predict qualitatively that both *G* and *S* can be optimized by bringing the HOMO (Figure 11(b)) or the LUMO level (Figure 11(c)) close to the chemical potential [187].

### 4.2. Single-Molecule Thermopower Measurements

Recent experiments have demonstrated that indeed thermopower of off-resonance single-molecule tunneling junctions behaves as expected from the theories [72]. The thermoelectric transport through a junction can be measured in a relatively simple way [193]. The method is based on the SPM break junction technique. Molecular bridges were formed by approaching a metal tip to a conductive substrate coated with a self-assembled monolayer of target molecules until the tip-substrate conductance reached a set value. To induce measurable thermoelectric voltage, a temperature gradient Δ*T* was applied by heating the substrate while keeping the tip at the ambient temperature. Subsequently, analogous to the single-molecule conductance measurements that monitor temporal change of the current flowing through the molecules under a constant bias voltage during junction stretching [14], thermopower was measured by recording the voltage Δ*V* required to cancel the thermal current between the tip and the substrate under the constant temperature gradient Δ*T* [193]. The thermoelectric voltage stayed at a constant voltage with relatively large fluctuations for a certain period and gradually decreased to zero as the electrodes were pulled apart, which manifested that Δ*V* is insensitive to the number of molecules bridging the electrodes. This is in sharp contrast to the electrical conductance of molecular junctions that increases with the number of current-carrying molecules. The thermopower could be deduced by exploring the linear dependence of Δ*V* on Δ*T*. Since the measured voltage includes thermoelectric power of Au electrodes, the junction thermopower was estimated through *S* = *S*_Au_ − Δ*V*/Δ*T*, where *S*_Au_ ∼ 1.94 μV/K is the Seebeck coefficient of bulk Au at 300 K [193].

One of the remarkable outcomes of the thermopower measurements is that whether a single-molecule junction is *n* type or *p* type can be judged from the sign of the thermoelectric voltage, as is usually done for bulk materials [189]. The theoretical expression of *S* indicates its dependence on the transmission curve line shape *∂T*(*E*)/*∂E*. From this, vanishingly small thermopower is expected when *E*_F_ aligns at the center of the HOMO-LUMO gap. On the other hand, when *E*_F_ is shifted closer to the HOMO (LUMO) level, *∂T*(*E*)/*∂E* takes a negative (positive) value and *S* is positive (negative); or conversely, positive *S* signifies that *E*_F_ is closer to HOMO, and *vice versa* [189]. Reddy *et al.* have found positive *S* for 1,4-benzenedithiols bridged between the Au electrodes, suggesting HOMO contributions to the electrical conductance [193]. This was in accordance with the results obtained by the gate modulation of molecular orbital levels. Furthermore, Widawsky *et al.* [194] positive and negative *S* for molecular junctions with Au-NH_2_ and Au-pyridine contacts, respectively. They could simultaneously measure the single-molecule conductance of these molecules, and quantitatively explained the results by the density functional theory calculations [194].

### 4.3. Molecular Length Dependence of Junction Thermopower

Molecular length dependence of the thermopower has also been explored for better understanding of thermoelectric transport in single-molecule junctions [195]. It has been well-established that an increase in the molecule length *L* leads to an exponential decay of the tunneling conductance as *G* = *G*_C_exp(−*β*_G_*L*), where *G*_C_ is the contact resistance and *β*_G_is the decay constant [14,23–27]. In contrast, the Seebeck coefficient of oligophenylenedithiols increased linearly with the number of phenyl rings [193,195]. Furthermore, oligophenylenediamines also showed similar *S* − *L* dependence but the intercept at *L* = 0 was low. From this observation, the length-dependent thermopower of the aromatic molecular junctions was described as *S* = *S*_C_ + *β*_S_*L*, where *S*_C_ denotes the contact thermopower that can be optimized by the choice of anchoring group and *β*_S_ is a coefficient. The linear increase in *S* was explained by the shrinking of the HOMO-LUMO gap with increasing the length of the molecule that reduces energy gap between the Fermi level and the frontier molecular orbital levels [195]. This clearly shows that the thermopower of single-molecule junctions is insensitive to the contact coupling, as predicted by Paulsson and Datta [189].

Interestingly, the junction-to-junction variations of the thermoelectric voltage were found to become more expansive with increasing *L* [196]. Moreover, the distribution of *S* was unaffected by adding a steric hindrance to reduce twisting angle between the phenyl rings of the oligophenylenedithiols. As *S* is less affected by *Γ* in the far from resonance regime, the distributions of the measured thermoelectric voltage can be attributed to a variation in the HOMO-Fermi level alignment presumably stemming from the difference in the contact motifs, the molecular orientations with respect to Au electrodes, and the interactions with nearby molecules [196]. The variation in the thermovoltage cannot be explained by the simplified model of Equation (4) [72], and continues to be an important issue to be resolved in future studies.

Although the thermopower is enhanced by increasing the molecular length [193,195], it also leads to exponential decrease of the conductance [196]. As such, the overall effect cannot improve the power factor. Therefore, along with the increased fluctuations of *G* and *S*, using a long molecular wire may not be a good choice for developing high-*ZT* single-molecule devices.

### 4.4. Potential and Challenges for High-ZT Single-Molecule Devices

Perhaps adjusting the Fermi level alignment *in situ* by gate modulation of the molecular orbital levels may be a suitable way to optimize the power factor. This approach has already demonstrated to be useful for improving the thermoelectric properties of Si nanowires [197]. An alternative strategy was also suggested by Baheti *et al.* [198]. They chemically tuned the HOMO level by dressing a molecule with donor or acceptor substituents. It was found that the Seebeck coefficient of *p*-type 1,4-benzenedithiols can be increased by adding electron donating methyl units, or decreased by decollating with electron withdrawing atoms such as F and Cl. These results can be interpreted by a shift of the HOMO level close to (away from) the Fermi level with the donor (accepter) groups. Though not yet verified, this method can in principle raise both *G* and *S* simultaneously as the conductance also increases with decreasing the HOMO-Fermi level energy gap for *p*-type molecules [198].

Much has been learned about thermoelectric transport in single-molecule junctions through the excellent experimental and theoretical studies. Recently, high thermopower has been reported for *n*-type single-fullerene molecules, and further improvements in the power factor can be expected with the chemical doping strategy [199]. For the prospect of high-*ZT* single-molecule thermoelectric devices, however, it is indispensable to evaluate and control the heat transport through single-molecule junctions and attain low *κ* [189]. Several studies have reported indirect estimations of the single-molecule thermal conductance [200,201]. Wang *et al.* [200] investigated the cross-plane thermal conductance of a self-assembled monolayer of alkanethiols by measuring the time required for a laser pulse heat to transfer through the molecules. The single-molecule thermal conductance of 50 pW/K was deduced from the obtained monolayer thermal conductance and the area density of the alkanethiols [200]. Another experiment examined heat dissipation in current-carrying benzenedithiol single-molecule junctions [201]. From the dissipated power and the estimated junction effective temperature, single-molecule junction thermal conductance was assessed to be 40 pW/K [201]. Although these experiments suggest that molecular junctions possess relatively low *κ*, heat transport in single-molecule bridges is largely unknown. Particularly, from a practical viewpoint, heat leakage by radiation and convection may become crucial issues considering the small size and the low thermal conductivity of single-molecule junctions. Future efforts should aim at direct measurements of thermal conductivity at the single-molecule level. This may be accomplished by developing nanostructures consisting of a heat source and a temperature sensor. One such system has already been fabricated for evaluating Joule heating in atomic-sized contacts [202].

## 5. Conclusions/Outlook

Owing to advances in nanotechnology, it has become more and more feasible to fabricate single-molecule junctions and test their properties in laboratories. The advent of the repeated break junction technique has revolutionized the understanding of electron transport in single-molecule junctions in the hopping, tunneling, and even ballistic regimes. IETS has now been used routinely to identify the presence and sometimes also the geometries of molecular bridges. Transition voltage spectroscopy has opened an avenue for quantitative insight into the Fermi alignment issue. These achievements, in conjunction with the progress in computational methods, have stimulated new molecular designs for controlling the electrical characteristics of metal-molecule-metal junctions, and now remarkable progress has been achieved in demonstrating proof of concept of single-molecule active electronic elements including diodes, switches, and transistors.

Now that individual organic molecules are found to be potential building blocks for logic and memory in nanoelectronics, the important future challenge in single-molecule electronics is to explore commercially viable fabrication methods and molecular functionalities. Operations of single-molecule electronic components were in many cases confirmed only in a cryogenic vacuum. Thus, it is essential to examine the device performance and durability of single-molecule junctions in a practical environment. Development of a cheap and reliable method for integration of the single-molecule logic and memory devices may also be required. While electromigration break junction is a promising technology for mass-production of functional molecular wire arrays, the poor production yields need further improvement. An alternative strategy for achieving high device yields is to exploit self-organized processes via sequential chemical reactions [203–206]. This fascinating bottom-up approach assembles molecular Lego blocks into a molecular bridge *in-situ* in an electrode nanogap by synthesis. Incorporating this interconnect scheme into electromigrated nanogaps would lead to better yields. Energy dissipation is another concern. It has been shown that single-molecule switches can be operated by nW-level electric power, which indicates very low power consumption. However, electron-vibration interactions in a current-carrying single-molecule junction cause a certain degree of power dissipation via local heating. Although energy dissipation *per* junction is expected to be marginal, large scale integration of molecular devices would lead to significant heating. There is still a long way to go before single-molecule electronic devices can be used in practical applications. Nevertheless, the field is progressing rapidly from both scientific and technological viewpoints encouraging further advances in single-molecule electronics.
